# Supplementary material for: Electrically modulated photothermal force microscopy for revealing molecular conformation changes during polarization switching at the nanoscale
Source: Nat Commun. 2025 Jul 21;16:6680. doi: 10.1038/s41467-025-61892-x (PMC12280132; doi:10.1038/s41467-025-61892-x)
Supplement: Supplementary file 1 — Supplementary Information [file 41467_2025_61892_MOESM1_ESM.pdf]

SUPPLEMENTARY INFORMATION

Electrically Modulated Photothermal Force Microscopy for Revealing Molecular Configuration Changes During Polarization Switching at the Nanoscale

Table of contents

Supplementary Note 1. Mechanisms and bias-induced phenomena in PFM and ePTFM

Supplementary Table 1. The settings for the IR pulse repetition rate in the experiment.

Supplementary Fig. 1. Details of the time-dependent variation of variations in the amplitude and electric bias.

Supplementary Fig. 2. Edge-on P(VDF-TrFE) IR spectra acquired on different platforms.

Supplementary Fig. 3. AFM-IR spectrum of edge-on P(VDF-TrFE).

Supplementary Fig. 4. Cross-sectional SEM image of the edge-on P(VDF-TrFE) film.

Supplementary Fig. 5. The IR-E loops at 1719 cm^{-1} of the PMMA film.

Supplementary Fig. 6. IR-E loop at 1076 cm^{-1} of edge-on P(VDF-TrFE).

Supplementary Fig. 7. Repeatability of butterfly patterns via the ePTFM.

Supplementary Fig. 8. Exclusion of the artifact of absorption peak shifting.

Supplementary Fig. 9. A full cycle spectrum experiment conducted on P(VDF-TrFE).

Supplementary Fig. 10. Domain writing at toroidal face-on P(VDF-TrFE).

Supplementary Fig. 11. Reflectance FTIR spectrum comparison of face-on and edge-on P(VDF-TrFE).

Supplementary Fig. 12. On-field IR–E loops of 1120 cm^{-1} and 1289 cm^{-1} in toroidal P(VDF–TrFE).

Supplementary Fig. 13. The ePTFM maps of the toroidal domain face-on P(VDF-TrFE).

Supplementary Fig. 14. Nanoscale characterization of ePTFM.

Supplementary Fig. 15. Nanoscale mapping of electrodriven evolution via ePTFM

Supplementary Fig. 16. Corresponding PLL frequency maps and IR phase maps of electrodriven ePTFM maps.

Supplementary References

Supplementary Note 1

Mechanism and bias-induced phenomena in PFM and ePTFM

PFM is essentially an atomic microscopy-based technique that observes bias-induced responses in a sample. As previously reported[1,2], the response that contributes to the PFM signal is not only an inverse piezoelectric response but also an additional contribution, including the electrostatic response, charge injection and Vegard strain. These additional contributions plague the target signal, giving a misleading picture of the ferroelectricity of the sample.

Considering that the deflection D of PFM contains surface displacement U and an electric force contribution of U_{ES}[1], the deflection D can express as

$$D = U+U_{ES} \tag{1}$$

where $U=QP^2$, Q represents the electrostrictive coefficient and P represents the polarization. For a polar organics P may include various contributions, here considering remanent polarization $\pm P_r$and induced polarization $\chi\varepsilon_0 V$, where χ is the polarizability, $\varepsilon_0$is the vacuum permittivity and $V = V_{DC}+V_{AC}\sin(\omega t)$ is the electric bias, in which V_{DC} is the direct current (DC) modulated bias applied to the tip, and V_{AC} is the amplitude of the tip AC probing bias. Thus, the polarization can be expressed as $P=\pm P_r+\chi\varepsilon_0(V_{DC}+V_{AC}\sin(\omega t))$. The surface displacement U can be written as:

$$\begin{aligned} U &= Q(\pm P_r+\chi\varepsilon_0 V)^2 \\ &= Q(P_r^2\pm 2\chi\varepsilon_0 P_r V+\chi^2\varepsilon_0^2 V^2) \\ &= Q(P_r^2\pm 2\chi\varepsilon_0 P_r V_{DC}\pm\chi\varepsilon_0 P_r V_{AC}\sin(\omega t) \\ &\quad +\chi^2\varepsilon_0^2 V_{DC}^2+2\chi^2\varepsilon_0^2 V_{DC}V_{AC}\sin(\omega t)+\chi^2\varepsilon_0^2 V_{AC}^2\sin^2(\omega t)). \end{aligned} \tag{2}$$

In addition, the electrostatic force between the tip/cantilever and sample is $F = \frac{C}{2}(V_{DC}\text{-}V_{SP}+V_{AC}\sin(\omega t))^2$, where C is the capacitance gradient between the tip/cantilever and sample, V_{SP} is the surface potential[3]. The corresponding displacement contribution U_{ES} can be expressed as follows:

$$U_{ES} = k^{-1}\frac{C}{2}\left(\left[(V_{DC}\text{-}V_{SP})^2+\frac{1}{2}V_{AC}^2\right]+2[V_{DC}\text{-}V_{SP}]V_{AC}\sin(\omega t)\text{ - }\left[\frac{1}{2}V_{AC}^2\cos(2\omega t)\right]\right), \quad (3)$$

where k is the effective contact stiffness. After the processing of the lock-in amplifier, the constants and signals with frequencies other than ω will be eliminated. Thus, the DC-modulated PFM amplitude is given as follows:

$$D_{amp}=\sqrt{[\pm 2Q\chi\varepsilon_0 P_r+2Q\chi^2\varepsilon_0^2 V_{DC}+k^{-1}C(V_{DC}\text{-}V_{SP})]^2 V_{AC}^2} \quad (4)$$

Because only high-order terms and constant terms can be filtered out, the first order term of V_{AC}, including the contribution of DC bias-induced polarization remains, as shown in the second term in the radical sign $2\chi^2\varepsilon_0^2 V_{DC}$. In addition, the electrostatic force contribution term $k^{-1}C(V_{DC}\text{-}V_{SP})$ also remains. Thus, the additional contributions of the DC bias cannot be separated by lock-in amplifier.

The SS-PFM (Switching Spectroscopy-PFM) experiment is taken as an example, which is used to measure PFM hysteresis loops. The piezoresponse can be obtained during the DC pulse by superimposing the measuring AC sinusoid, or, alternately, immediately after each DC pulse. The DC voltage is varied in a series of voltage pulses with varying magnitudes. These two situations are named “on-field” and “off field”[1]. The purpose of off-field observables was to minimize the electrostatic contributions bring by DC bias. The selection of off-field results for analysis is a widely used routine. This occurred because the effect of the DC bias generates a bending

response that cannot distinguished from AC bias induced response. However, by designing the testing procedure, the current PFM attempts to avoid relevant measurements in the presence of DC voltage. However, immediately after its removal, the DC voltage may still result influence including injected charge and redistribution of ionic and electronic species, which will also construct a bias electric field to have a similar interference effect. Moreover, it is also of high interest to study the voltage-dependence of the bias-induced polar state, especially for volatile polar organics whose polar state cannot be maintained after removing the DC bias.

However, the utilization of pulsed infrared radiation can get rid of electrostatic interference or charged species redistribution and accumulation induced by DC electric bias. When a DC bias is applied to the sample, it produces two major influences on ePTFM. First, DC bias will induce reorientation of a specific chemical structure and the corresponding transition dipole moment, which will lead to changes in IR absorption and detected by ePTFM. Secondly, DC bias will also induce additional contributions such as the accumulation of charged species et.al. These additional contributions normally have different IR absorption characteristic frequencies to the polar bonds and groups we observed. Thus, they would not affect the results because of the specificity of the characteristic wavenumber. Moreover, in ePTFM, the AC electric field caused by IR light reaches the terahertz region, which does not share the same frequency of lock-in amplifier’s reference signal. Since no AC voltage signal is coupled with spurious effects as in Eq (4), the DC bias-induced additional contribution can be eliminated using pulsed IR.

Supplementary Tables

Supplementary Table 1. The settings for the IR pulse repetition rate in the experiment.

Corresponding Figures	Setting of IR pulse frequency
Figure 1c	201.69 kHz
Figure 2e	178.73 kHz
Figure 2f	198.32 kHz
Figure 3c	201.91 kHz
Figure 3f	173.72 kHz and 201.91 kHz
Figure 3f and 3g	196.48 kHz
Figure 4b~h	196.81Khz

Supplementary Figures

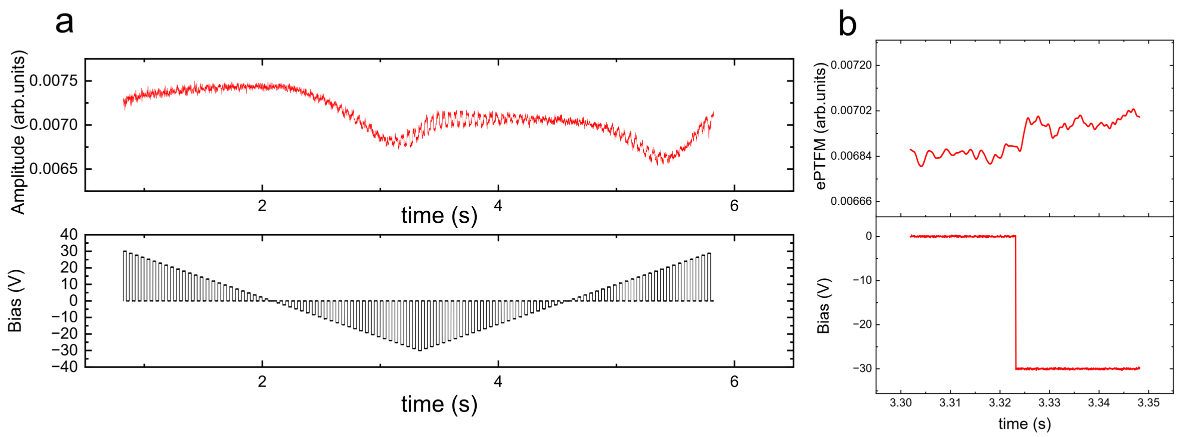

Supplementary Fig. 1. Details of the time-dependent variations in the amplitude and electric bias. a IR absorption is recorded under a triangle-square bias. Amplitude trace (red), which indicates the IR absorption, varied with the modulated bias. **b** Corresponding zoom-in image shows ePTFM signal before and after applied 30V electric bias.

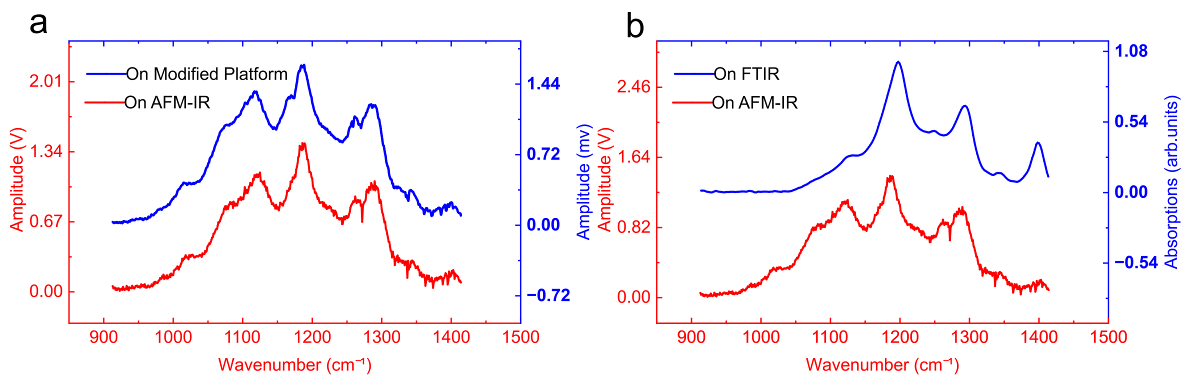

Supplementary Fig. 2. Edge-on P(VDF-TrFE) IR spectra acquired on different platforms. a Comparison between the modified ePTFM platform and the original AFM-IR proves that the basic functionalities are unaffected. **b** Comparison of the FTIR and original AFM-IR platform.

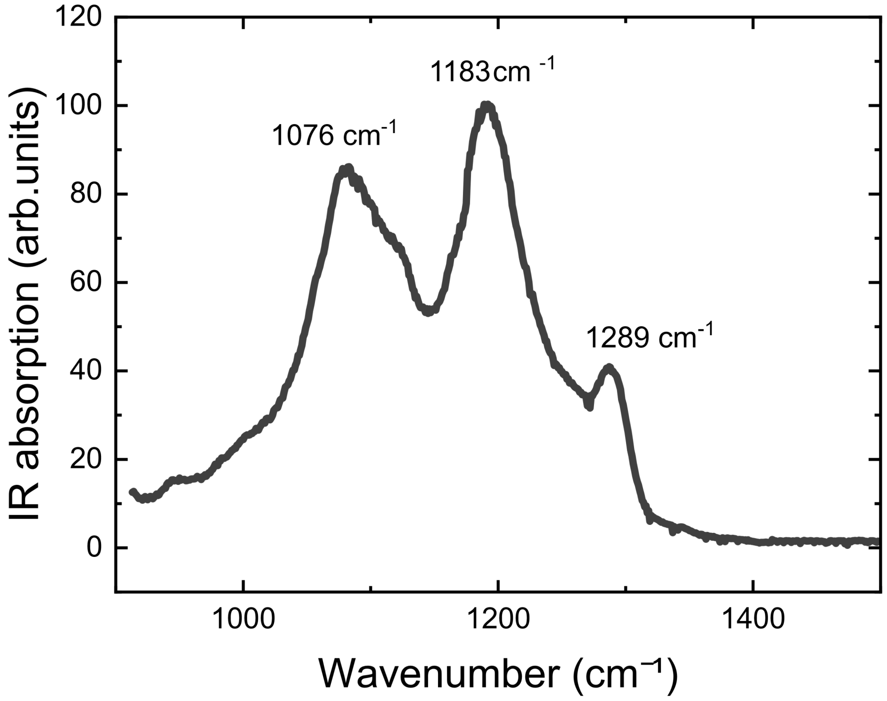

Supplementary Fig. 3. AFM-IR spectrum of edge-on P(VDF-TrFE). The results show edge-on P(VDF-TrFE) has obvious absorption peaks at 1076 cm^{-1}, 1189 cm^{-1}, and 1289 cm^{-1}.

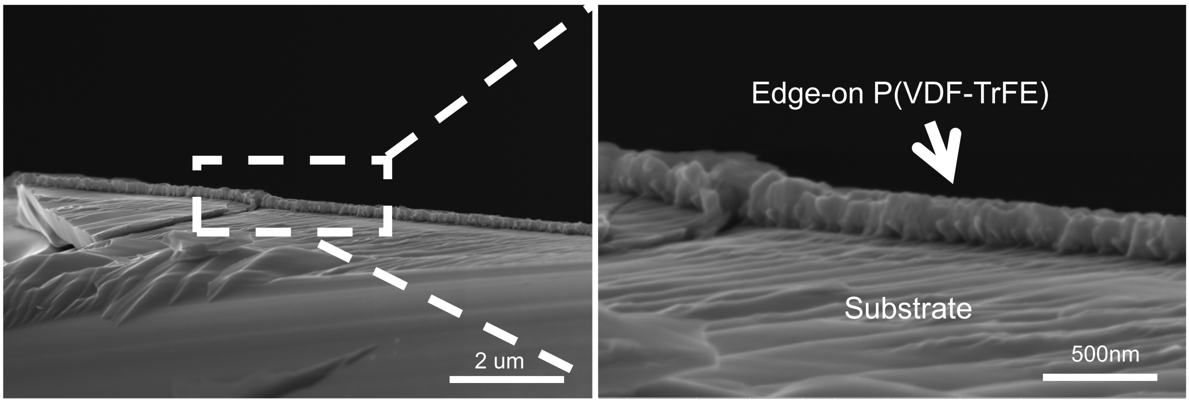

Supplementary Fig. 4. Cross-sectional image SEM of the edge-on P(VDF-TrFE) film. The thickness of the lamellae is approximately 200 nm. The white lines denote scale bars.

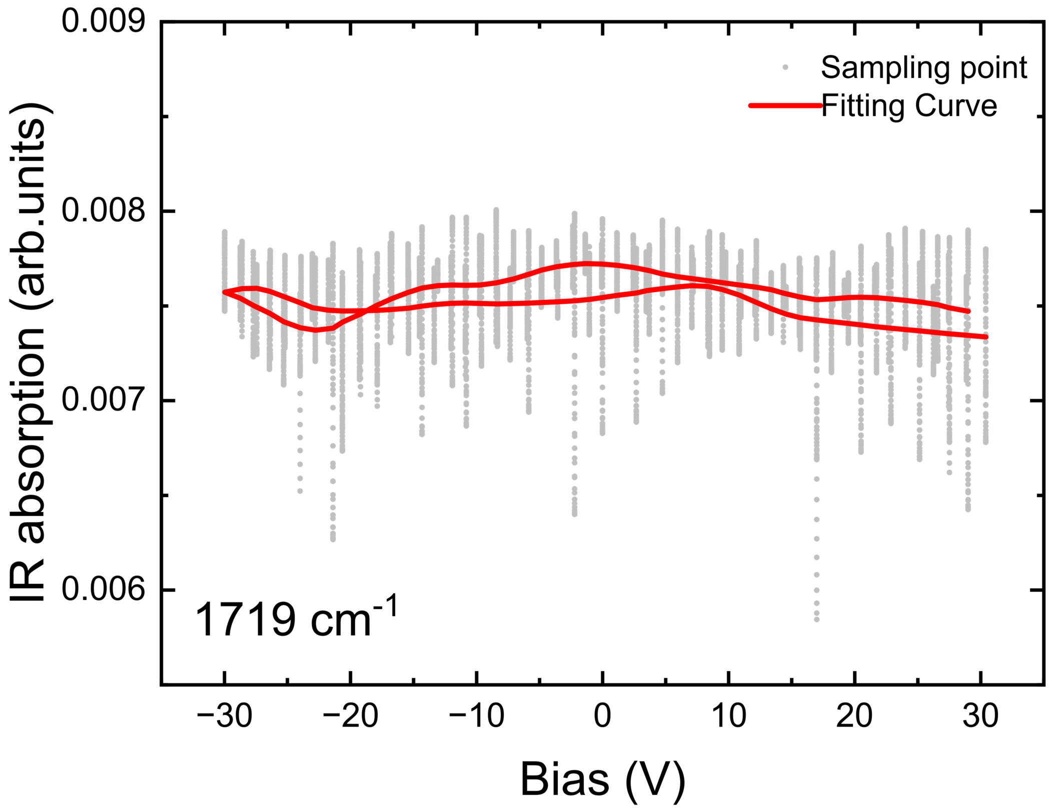

Supplementary Fig. 5. The IR-E loops at 1719 cm^{-1} of the PMMA film. 1719 cm^{-1} was assigned to the C=O stretching vibration. The PMMA film was spin-coated on a silicon substrate with a Pt coating. The grey dots correspond to the distribution of 64 sampling data points of cantilever amplitude at each off-field stage. The red solid line corresponds to the fitting curve according to the average value of the sampling points. Curve fitting was conducted via local regression methods.

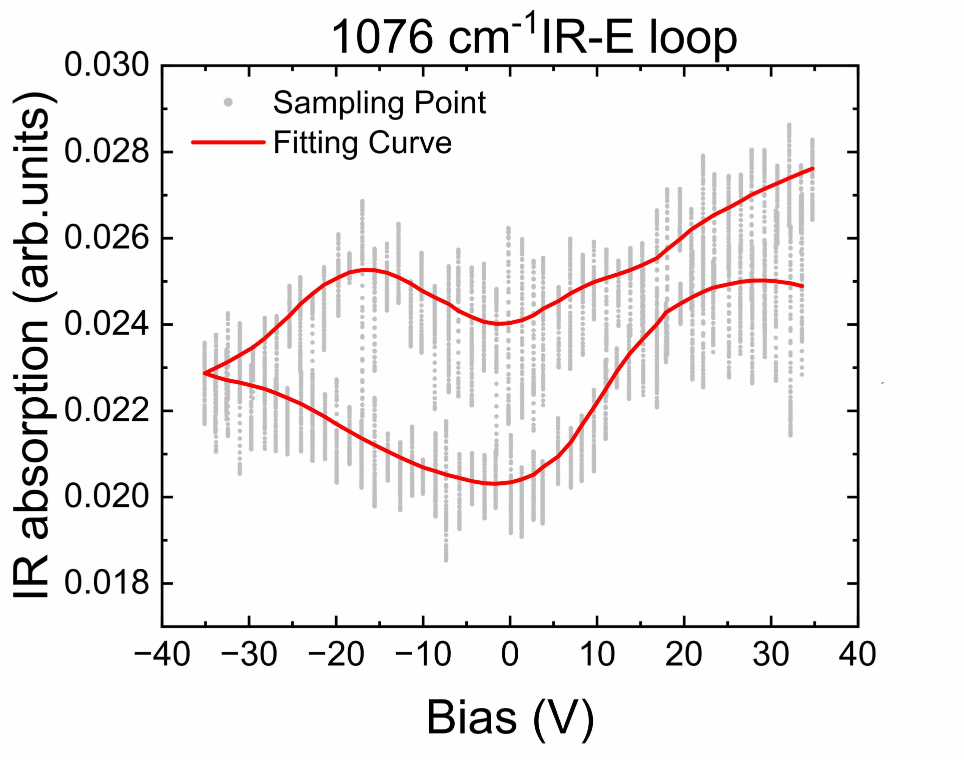

Supplementary Fig. 6. IR-E loop at 1076 cm^{-1} of edge-on P(VDF-TrFE). 1076 cm^{-1} is assigned to the vibration of the C–C stretch and has a dipole derivative direction along the polymer chain. This result clearly shows that there is no butterfly hysteresis.

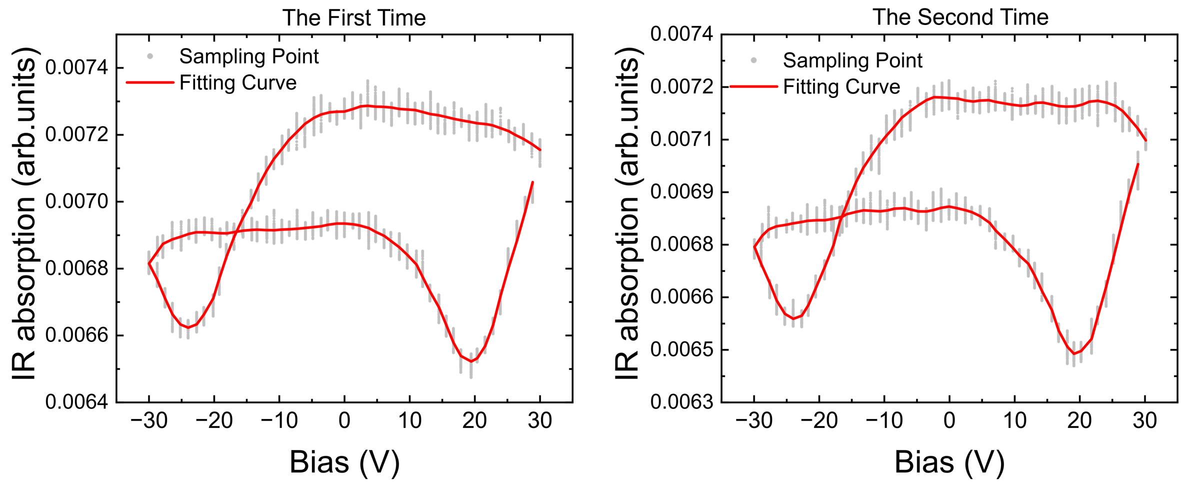

Supplementary Fig. 7. Repeatability of butterfly patterns via ePTFM. IR-E loops at 1289 cm^{-1} were obtained continuously under triangular square electric modulated bias.

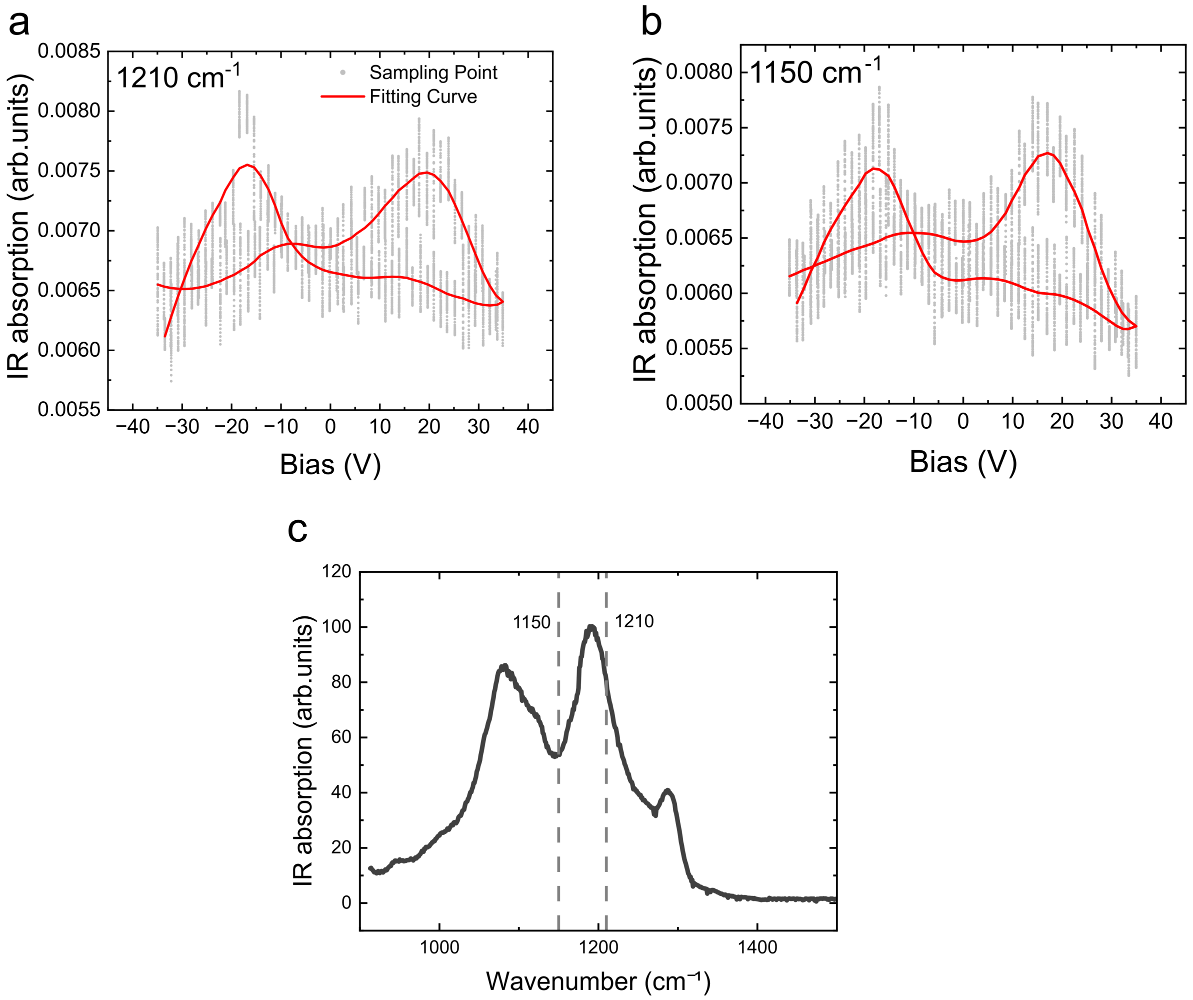

Supplementary Fig. 8. Exclusion of the artifact of absorption peak shifting. a IR-E loops at 1210 cm^{-1}. **b** IR-E loops at 1150 cm^{-1}. **c** AFM-IR amplitude of edge-on P(VDF-TrFE). To exclude interference from the shift in the absorption peak under electric bias, the IR-E loops at 1150 cm^{-1} and 1210 cm^{-1}, which were sandwiched with the 1183 cm^{-1} peak, were obtained and compared. The opposite trend of the IR-E loops occurs if the butterfly pattern is caused by the shifting of absorption peak. However, the two IR-E loops show the same variation trend, ruling out the shifting of the characteristic peak during the IR-E measurement.

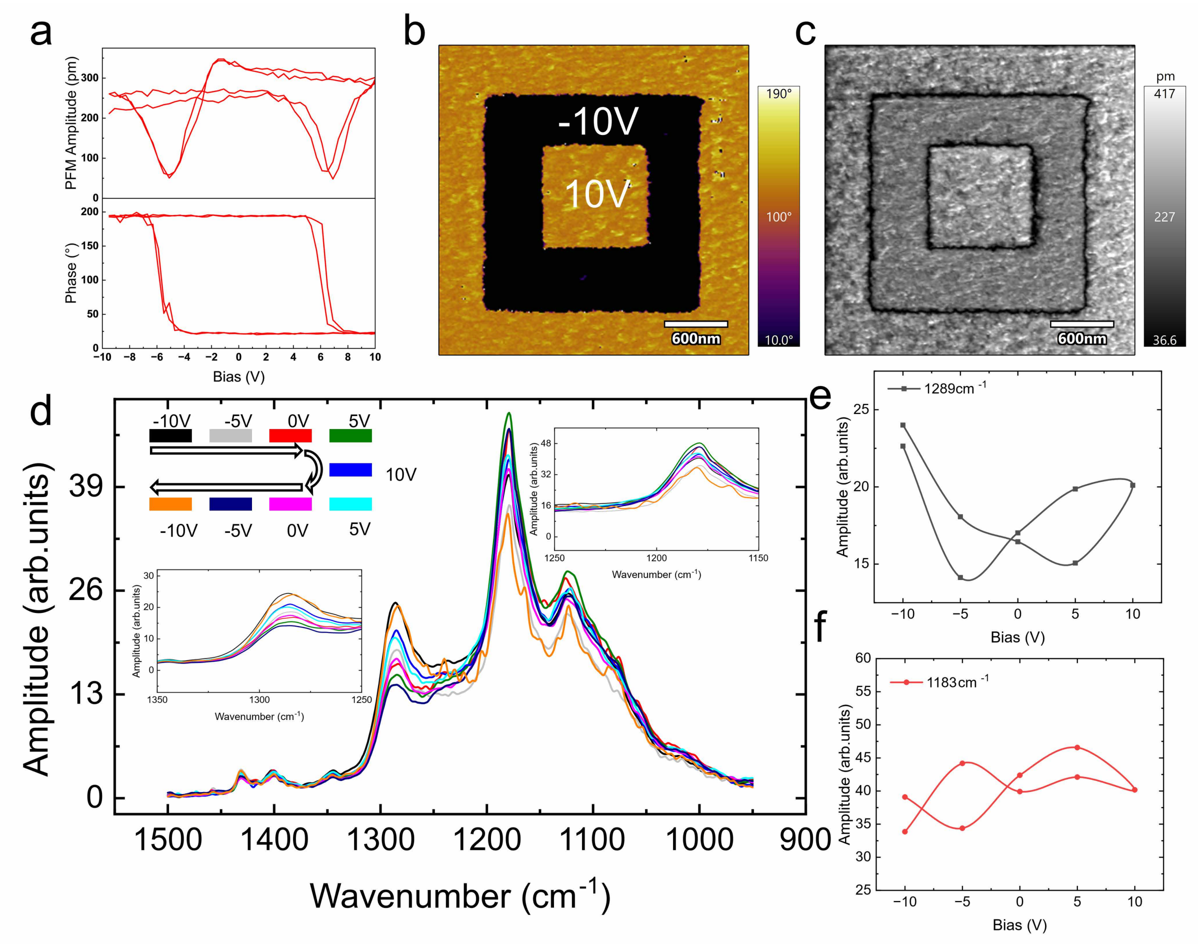

Supplementary Fig. 9. A full cycle spectrum experiment conducted on P(VDF-TrFE). The PFM hysteresis loops (a) exhibit distinct ferroelectric behavior, while OOP-plane PFM phase mapping results (b), and the corresponding amplitude results (c) further confirm the ferroelectricity of the edge-on P(VDF-TrFE) sample. Notably, the switching voltages are observed at approximately 5V. The spectrum (d) was collected under a full cycle DC bias, ranging from -10 V to 10 V and then back to -10 V. The insets provide a closer examination of the spectrum at the peaks of 1289 cm-1 and 1183 cm-1. Additionally, e and f show the evolution of the amplitude at 1289 cm-1 and 1183 cm-1 with changing bias, respectively. These results are consistent with the IR-E loops result in our manuscripts, offering a consistent understanding of the ferroelectric evolution characteristics. The P(VDF-TrFE) film was fabricated using the

same methods described in the manuscript, but with a lower concentration solution of 1% w/v and the omission of self-assembly step.

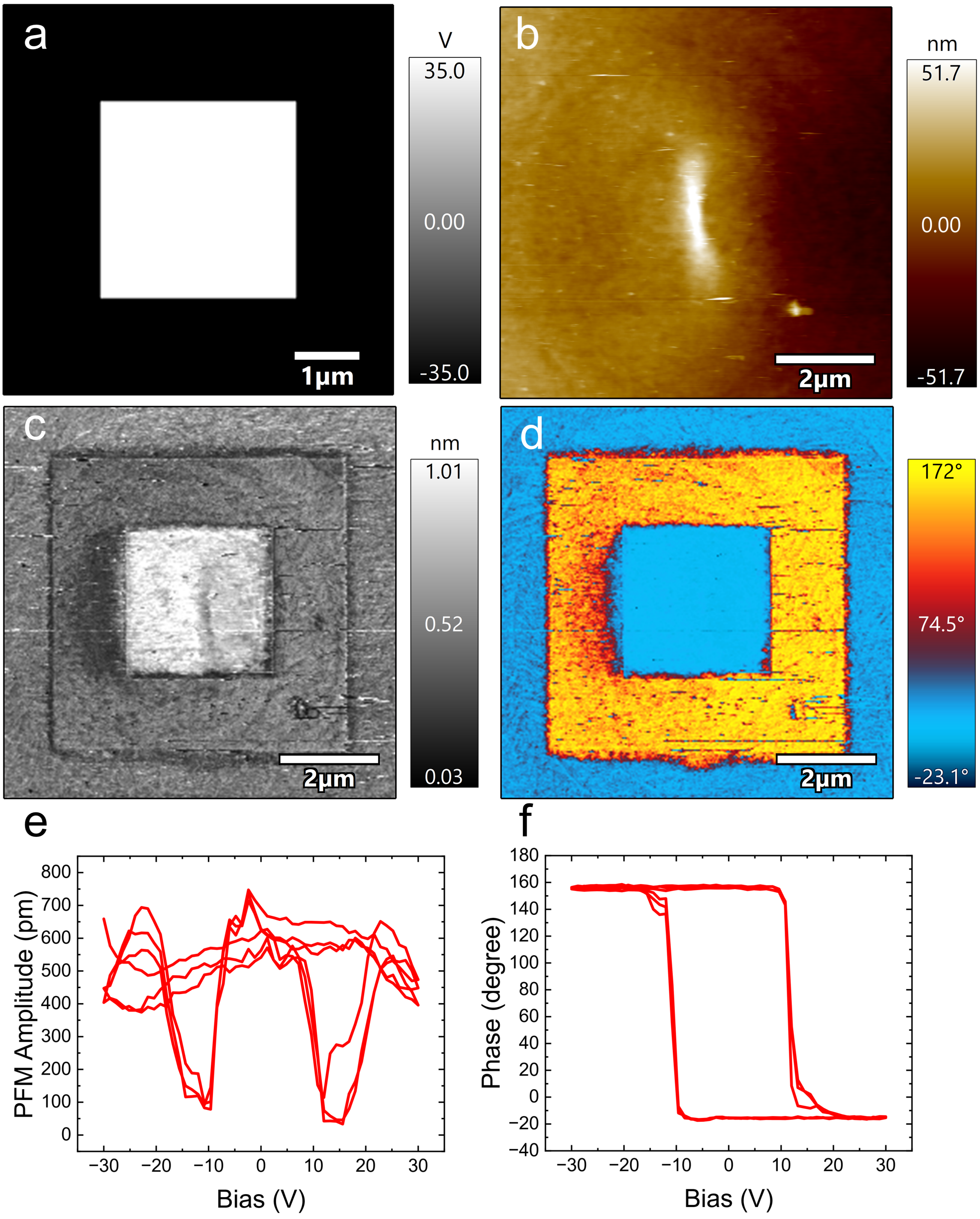

Supplementary Fig. 10. Domain writing at toroidal face-on P(VDF-TrFE). a Applied bias distribution. **b** Topology of face-on P(VDF-TrFE). **c** Amplitude of the same region. **d** PFM phase distribution of the same region. The square pattern still existed after several days. **e** Amplitude variation in the PFM-switching spectrum of toroidal face-on P(VDF-TrFE). **f** Phase variation in the PFM-switching spectrum of toroidal face-on

P(VDF-TrFE). The hysteresis loops in PFM confirm the out-of-plane ferroelectricity. The white lines in a, b, c, and d denote the scale bars.

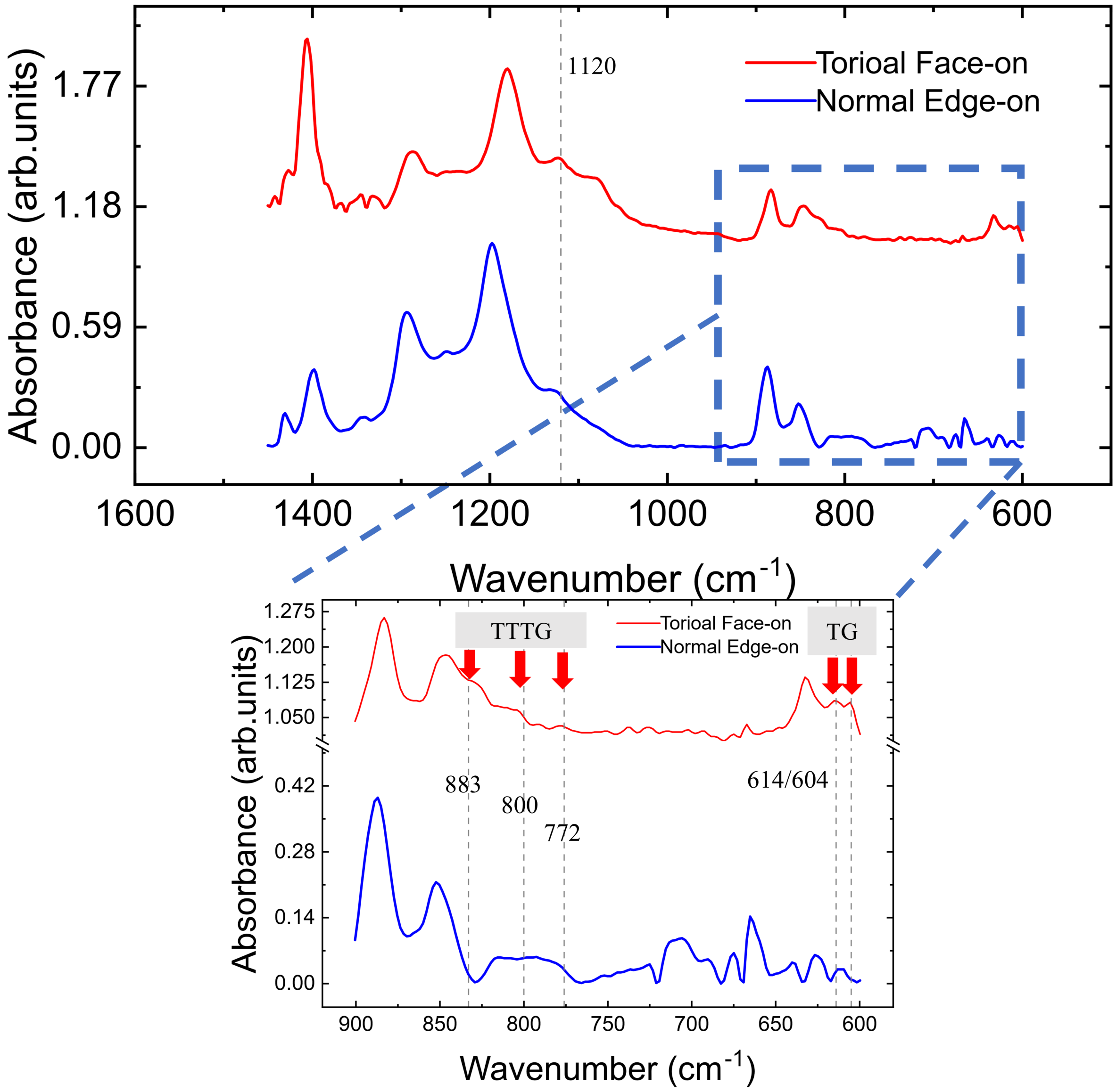

Supplementary Fig. 11. Reflectance FTIR spectrum comparison of face-on and edge-on P(VDF-TrFE). The detailed views of the spectral changes are shown below. The red arrows show the trends of changes in the characteristic peaks. The intensities of the characteristic bands corresponding to the TTTG conformation[4-6] near 833 cm^{-1} 800 cm^{-1} and 772 cm^{-1} were observed. The short sequence of TG at 614/604 cm^{-1} in double band[5] was also observed.

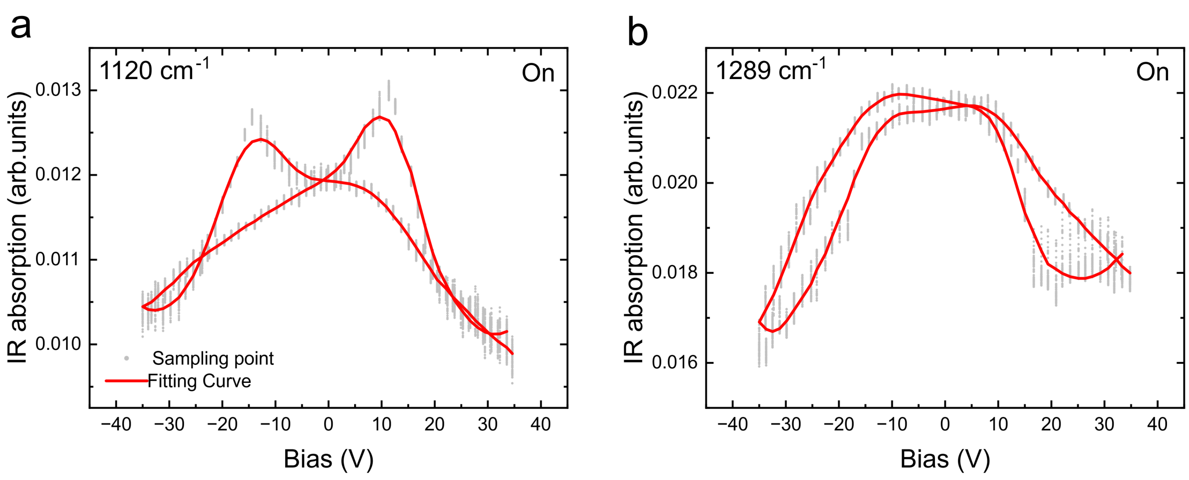

Supplementary Fig. 12. On-field IR-E loops of 1120 cm^{-1} and 1289 cm^{-1} in toroidal P(VDF-TrFE). a Off-field IR-E loop of 1120 cm^{-1} with an obvious butterfly pattern. **b** Off-field IR-E The grey dots correspond to 64 sampling data points of cantilever amplitude at each on-field stage.

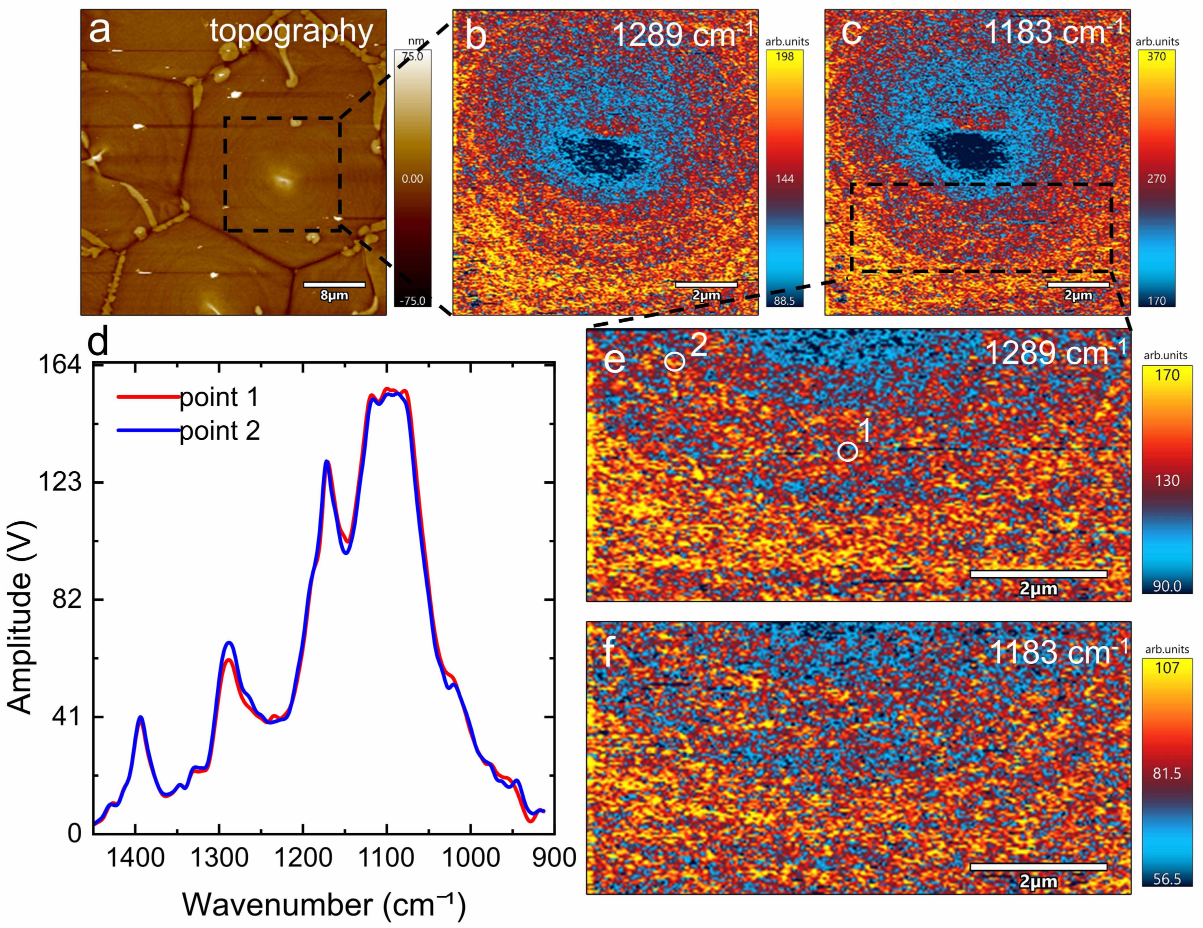

Supplementary Fig. 13. The ePTFM mappings of the toroidal domain face-on P(VDF-TrFE). a Topography of face-on P(VDF-TrFE). **b** The ePTFM mapping of the absorption at 1289 cm^{-1}. **c** ePTFM mapping of the absorption at 1183 cm^{-1}. **d** Comparison of the local IR spectra of different points marked in e. If the variation of mappings was caused by mechanical effect, the intensity of spectrum may increase or decrease. However, the spectrum shows that only the corresponding intensity of 1289 cm^{-1} changes, which shows that the distribution of the IR mapping was not due to noise or mechanical effects. **e** Zoomed-in 1289 cm^{-1} IR mapping of b. **f** Zoomed-in 1183 cm^{-1} IR mapping of c. The ePTFM mapping was conducted under p-polarization. For P(VDF-TrFE) with toroidal polar structures, the transition dipole moment is predominantly distributed within the plane (Please refer to Figure 3 and the

corresponding discussion). Thus, even though the in-plane polarization of toroidal P(VDF-TrFE) was varied at different locations, it’s reasonable that the maps do not reflect corresponding contract and presented nearly the same spatial distribution.

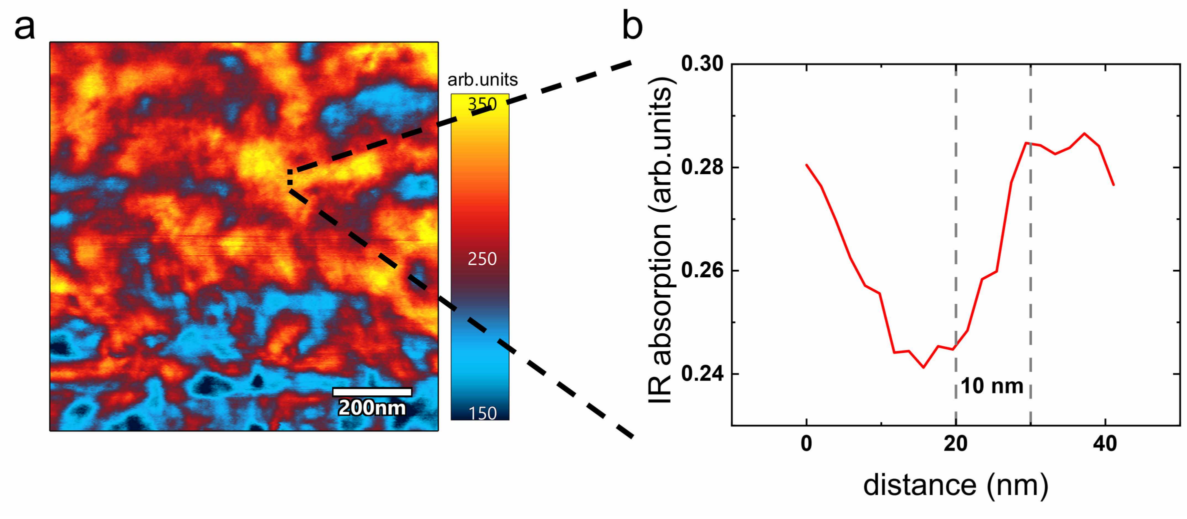

Supplementary Fig. 14. Nanoscale characterization of ePTFM. a ePTFM mapping at 1120 cm^{-1} under 15 V bias. The white lines denote scale bars. **b** Section profile at the white dashed line in a, showing the nanoscale ability of ePTFM.

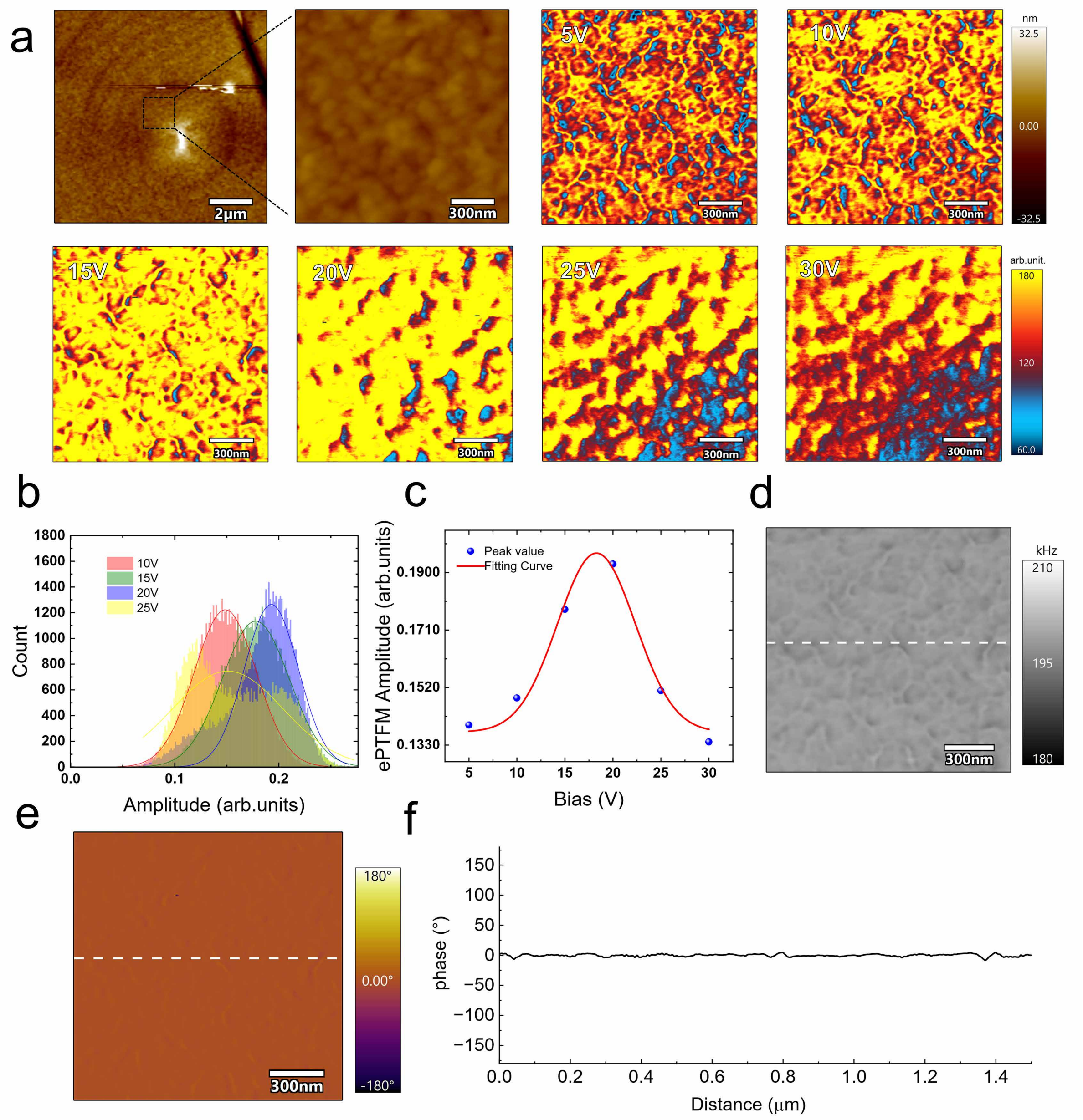

Supplementary Fig. 15. Nanoscale mapping of electrodriven evolution via ePTFM. a Corresponding nanoscale ePTFM maps of 1120 cm^{-1} absorption at 5 V, 10 V, 15 V, 20 V, 25 V, and 30 V. To demonstrate the intrinsic evolution, the mapping results use the original data without flattening or further processing. The upper color box applies to the height image, whereas the lower color box applies to the other images. **b** Histograms of the 10 V, 15 V, 20 V, and 30 V ePTFM mappings. The solid line represents the fitting curve of each histogram. **c** Corresponding mean value of the ePTFM signal. **d** Corresponding PLL frequency maps in ePTFM maps at 15V. **e** Corresponding IR phase maps result in ePTFM maps at 15V. **f** Cross-sectional profile corresponding to the dashed white line in d.

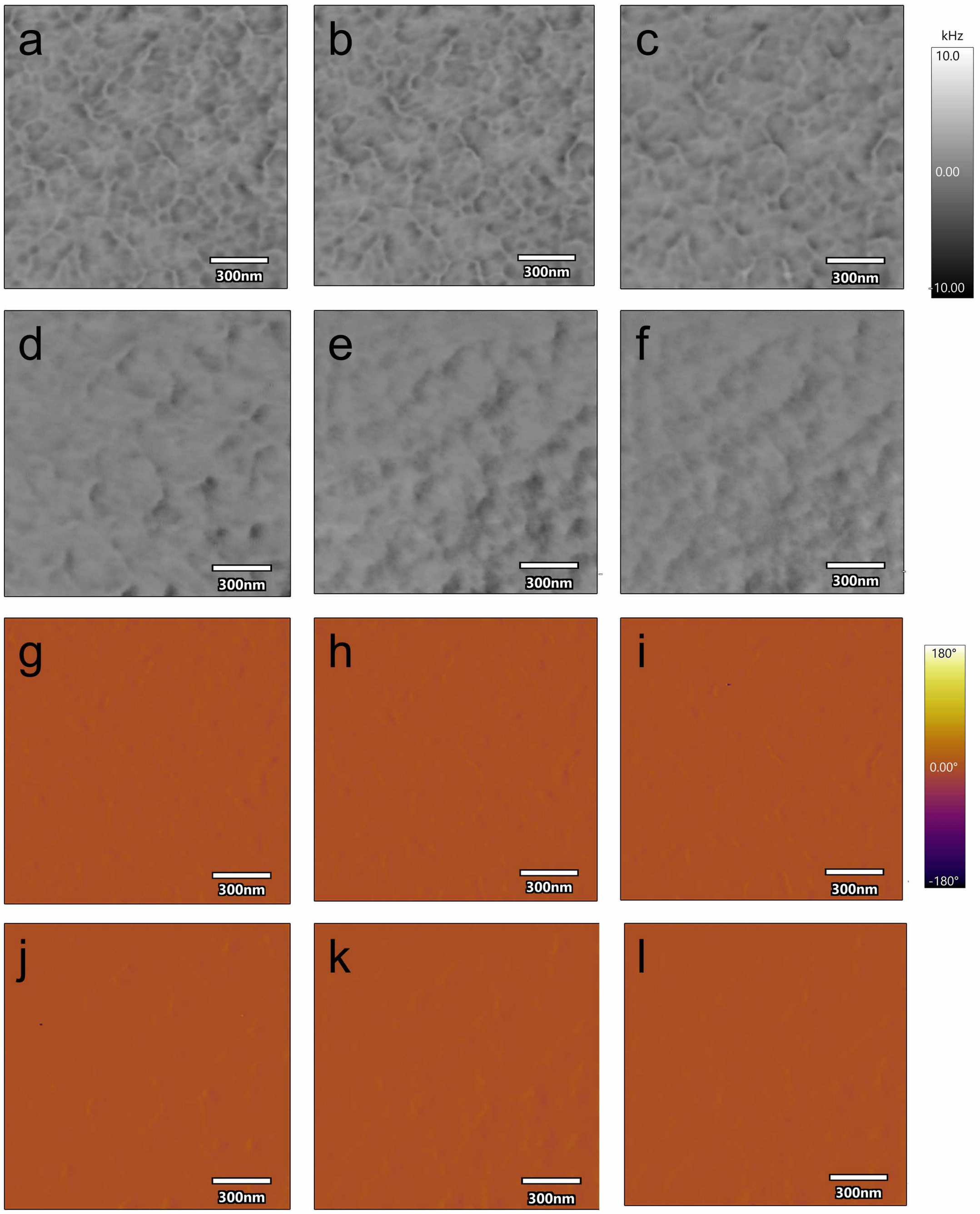

Supplementary Fig. 16. The corresponding PLL frequency maps and IR phase maps of electrodriven ePTFM maps. a~f The corresponding PLL frequency maps at 5 V, 10 V, 15 V, 20 V, 25 V, and 30 V, respectively. **g~l** The corresponding IR phase maps at 5 V, 10 V, 15 V, 20 V, 25 V, and 30 V, respectively.

Supplementary references:

1. Vasudevan, R. K., Balke, N., Maksymovych, P., Jesse, S. & Kalinin, S. V. Ferroelectric or non-ferroelectric: why so many materials exhibit "ferroelectricity" on the nanoscale. *Appl. Phys. Rev.* **4,** 021302 (2017).
2. Killgore, J. P., Robins, L. & Collins, L. Electrostatically-blind quantitative piezoresponse force microscopy free of distributed-force artifacts. *Nanoscale Adv.* **4,** 2036-2045 (2022).
3. Kim, Y. et al. Nonlinear phenomena in multiferroic nanocapacitors: joule heating and electromechanical effects. *ACS Nano* **5,** 9104-9112 (2011).
4. Martins, P., Lopes, A. C. & Lanceros-Mendez, S. Electroactive phases of poly(vinylidene fluoride): determination, processing and applications. *Prog. Polym. Sci.* **39,** 683-706 (2014).
5. Zhu, Y. et al. Operando investigation of the molecular origins of dipole switching in P(VDF-TrFE-CFE) terpolymer for large adiabatic temperature change. *Adv. Funct. Mater.* **34,** 2314705 (2024).
6. Bachmann, M. A., Gordon, W. L., Koenig, J. L. & Lando, J. B. An infrared study of phase-III poly(vinylidene fluoride). *J. Appl. Phys.* **50,** 6106-6112 (1979).
